# Social cognition

**DOI:** 10.1098/rstb.2008.0005

**Published:** 2008-02-21

**Authors:** Chris D Frith

**Affiliations:** 1 Wellcome Trust Centre for Neuroimaging, University College London12 Queen Square, London WC1N 3BG, UK; 2 Center for Functionally Integrative Neuroscience, University of Åarhus8000 Åarhus C, Denmark

**Keywords:** social, cognition, signals, meta-cognition, culture, observation

## Abstract

Social cognition concerns the various psychological processes that enable individuals to take advantage of being part of a social group. Of major importance to social cognition are the various social signals that enable us to learn about the world. Such signals include facial expressions, such as fear and disgust, which warn us of danger, and eye gaze direction, which indicate where interesting things can be found. Such signals are particularly important in infant development. Social referencing, for example, refers to the phenomenon in which infants refer to their mothers' facial expressions to determine whether or not to approach a novel object. We can learn a great deal simply by observing others. Much of this signalling seems to happen automatically and unconsciously on the part of both the sender and the receiver. We can learn to fear a stimulus by observing the response of another, in the absence of awareness of that stimulus. By contrast, learning by instruction, rather than observation, does seem to depend upon awareness of the stimulus, since such learning does not generalize to situations where the stimulus is presented subliminally. Learning by instruction depends upon a meta-cognitive process through which both the sender and the receiver recognize that signals are intended to be signals. An example would be the ‘ostensive’ signals that indicate that what follows are intentional communications. Infants learn more from signals that they recognize to be instructive. I speculate that it is this ability to recognize and learn from instructions rather than mere observation which permitted that advanced ability to benefit from cultural learning that seems to be unique to the human race.

## 1. What is social cognition?

As currently used, the term ‘cognition’ refers to the many different processes by which creatures understand and make sense of the world. The term does much the same work as was previously done by the term ‘information processing’ and is strongly influenced by developments in computing beginning in the 1940s. Perception, attention, memory and action planning would all be examples of cognitive processes. All these processes are important in social interactions and the study of information processing in a social setting is referred to as social cognition. ‘The goal of social cognition is to provide mechanistic, process-oriented explanations of complex social phenomena’ (Winkielman & Schooler in press). In this paper, I want to consider whether there are aspects of cognition that are specifically social and specifically human.

When we interact with the environment, psychologists have traditionally started from the input. Signals arising from the environment impinge upon us. Sensations are detected by our sense organs such as the eyes. The sensations (e.g. light of a certain wavelength) are turned into perceptions (e.g. the colour of the fruit) on the basis of prior knowledge and current context. Then, decisions are made about what should best be done in response to these perceptions (e.g. Is the fruit ripe? Should I eat it?). Actions are planned and finally output is initiated in the form of motor movements (e.g. grasping the fruit). Within this general framework of stimulus and response, we can have a subset of processes concerned with social stimuli (e.g. reading facial expressions), social decisions (Should I trust this person?) and social responses (making facial expressions)

### (a) Mirror systems and social stimuli

There is currently much interest in mirror systems in the brain. A mirror system is defined as a collection of brain regions that are active when we do or experience something ourselves, and also when we observe someone else doing the same thing or having the same experience. The concept originated from the observation of neurons in the frontal cortex of the monkey, which respond when the monkey performs a specific action (e.g. picking up a peanut) and also when the monkey observes someone else performing the same action (Rizzolatti & Craighero 2004). These neurons are now known as mirror neurons. In humans, mirror systems have been identified for emotion (Singer *et al*. 2004; Botvinick *et al*. 2005) and touch (Blakemore *et al*. 2005) as well as action (Rizzolatti & Craighero 2004). It seems plausible that systems that link actions and experiences in the self with actions and experiences in others are likely to have an important role in social cognition. We might even define one class of social stimuli as those stimuli that activate mirror systems. However, we also need to consider what value such stimuli might have in helping us to navigate successfully through the social world.

## 2. Social stimuli that tell us about the world

### (a) Avoiding danger

Physical disgust is an instinctive emotional reaction to sights and smells which helps us to avoid food poisoning or infection. The sight of someone with an expression of disgust is a signal that they are in contact with something that we should avoid. There is a mirror system for disgust (Wicker *et al*. 2003). When we see a disgusted face, we feel disgusted ourselves and may automatically take avoiding action before we consciously recognize the expression or discover the cause of the disgust.

We can tell a similar story for fear for which there is also evidence of a mirror system (Morris *et al*. 1996). The sight of a fearful face is a signal that there is something for us to be afraid of, and, as with disgust, elicits fear in the observer. In the case of fearful expressions, there are several experiments demonstrating that the presentation of a fearful face elicits physiological signs of fear in observers, even when they are not aware of seeing the face (e.g. Whalen *et al*. 1998). Elizabeth Phelps and her colleagues (Olsson & Phelps 2004) have shown that people can learn to fear an object (such as a blue square) simply by watching someone else being conditioned to fear that object, because each time the blue square is presented the person observed receives a painful shock. This learning by observation occurs even when the conditioned stimulus (the blue square) is masked and the observer is unable to report when this stimulus occurs. The most probable mechanism underlying this subliminal learning by observation is classical Pavlovian conditioning. We know (Ohman & Mineka 2001) that someone can be conditioned when the conditioned stimulus (CS, e.g. a blue square) is presented subliminally and followed by a shock (the unconditioned stimulus, US). In the case of subliminal learning by observation, the unconditioned stimulus is the sight of the face of the person in pain, since this stimulus elicits ‘pain’ in the observer.

Disgust and fear are signals emanating from other peoples' faces that indicate that there is something in their immediate environment to be avoided. However, a face can also supply a signal that the person should be avoided. When confronted with unknown people, there is a high level of inter-subject agreement that certain faces look trustworthy, while others look untrustworthy. The presentation of untrustworthy faces elicits activity in the amygdala, a physiological sign that avoiding action should be taken. This seems to be an automatic response, since it occurs whether subjects are explicitly asked to rate faces for trustworthiness or are attending to an irrelevant aspect of the faces such as sex (Winston *et al*. 2002). Unlike readings of the facial expression of fear, our reading of the facial expression of untrustworthiness seems to be an example of prejudice. While there is considerable agreement between people as to what an untrustworthy face looks like, there is no evidence for any validity for this reading. Presumably our idea about what an untrustworthy face looks like has been acquired through culture. Yet, this cue is still processed automatically, like signals of fear.

### (b) Learning which things are nice and which are nasty: social referencing

Closely related mechanisms can explain the phenomenon of social referencing (Feinman *et al*. 1992). Learning about the world from other people is particularly important during infancy when so much is novel. Confronted with a novel object or situation, the infant will look at his or her mother. A smile will cause the infant to approach while a frown will elicit avoidance. In this way, the infant can learn about a basic property of things in the world: whether they are nice or nasty. However, the infant does not learn about anything or from anyone. Through evolutionary history, the brain is pre-prepared to learn more rapidly about threatening stimuli, such as snakes (Mineka & Ohman 2002). Infant monkeys rapidly learn to fear snakes by observing fear in a model, but do not learn to be afraid of a flower by such observation (Cook & Mineka 1989). Initially, human infants learn about the world from observing their mothers, rather than strangers (Zarbatany & Lamb 1985). However, at 14 months, they will learn from a familiarized stranger (Klinnert *et al*. 1986) and by 24 months strangers are used as a source for learning (Walden & Kim 2005).

Like all signals, those used in social referencing are inherently ambiguous. The default assumption is that signs of fear tell us that an object is nasty and should be avoided. But, instead of telling us about the object, the signal could be telling us about the person showing fear. Perhaps this person has an abnormal attitude to this object, such as a phobia. Fourteen-month-old infants do not seem to make this distinction. They behave as if signals only tell us about the object, not about the person signalling (Gergely *et al*. 2007). However, by the age of 18 months, infants are able to make this distinction. I shall come back to this problem of the signal and the signaller in §6.

### (c) Finding locations of interest

In order to learn, from his or her mother's expression, whether an object is nice or nasty, the infant must know which object his or her mother is looking at. The infant can do this by taking into account the eye gaze direction of his or her mother. We are very accurate at gauging eye gaze direction (Anstis *et al*. 1969). Furthermore, when we see a person with averted gaze, we tend, automatically, to look at the place at which they are looking. We expect there to be something of interest at this location. Bayliss & Tipper (2006) used eye gaze direction in various faces as cues in a spatial attention task. Some faces gave valid cues, some neutral and some invalid cues. There is known to be a strong validity effect of eye gaze cues (Driver *et al*. 1999). Subjects are much slower to identify objects that appear in the opposite location to that indicated by the eye gaze direction (invalid cue). Bayliss & Tipper found that this effect occurred even for faces that consistently looked in the wrong direction. Subjects seemed to be unaware of these contingencies. However, after testing, the subjects rated the faces giving invalid spatial cues as appearing less trustworthy. We see two social processes here that seem to be largely automatic and unconscious. First, the shift of attention that is caused by observing someone's eye gaze direction and, second, the learning about how helpful people are from their behaviour.

## 3. Social responses mirror social stimuli

From a stimulus–response perspective, social cognition is very symmetrical. One person's stimulus is another person's response. This symmetry is most obvious in the various examples of the mirror system. I observe your fearful expression (a social stimulus), which causes me to make a fearful expression (a social response). Social interactions typically involve chains of such stimuli and responses. For example, Keltner & Buswell (1997) consider the case of the expression of embarrassment. Our protagonist has committed a social *faux pas* and his companions express anger. He responds by expressing embarrassment. His companions express sorrow as an empathic response to his discomfort. His appeasement has worked and every one expresses happiness.

In this example, the facial movements made by the participants not only express emotions, but they also have a communicative role. For our protagonist's appeasement to work, it is enough that he expresses the emotion of embarrassment. He does not have to feel it. Evidence that the expression of emotions has a strong communicative role comes from the observation that the presence of others markedly influences the magnitude of facial responses (Parkinson 2005). For example, Bavelas *et al*. (1986) demonstrated that an observer shows much greater signs of sympathy (via motor mimicry) when the person they are watching is in eye contact with them. The cynic might conclude that these facial movements do not reflect sympathy, but rather reputation management. The sender wishes to persuade the observer that he, the sender, is a sympathetic person. However, this interpretation requires that the expression of sympathy should be deliberate and consciously controlled. It is my feeling, and this opinion must be tested experimentally, that most of these facial movements are automatic and occur without conscious control. In §6*b*, I will consider those special signals that are deliberately communicative.

### (a) Mirroring responses

When we interact with someone we often mirror each other's movements and mannerisms, leading to synchronized leg-crossing, nodding and so on. This is known as the chameleon effect (Chartrand & Bargh 1999). We are unaware of this mirroring, but, when it occurs, it creates the feeling that we have good rapport with each other. This good feeling is not just directed at our companion, but to the world in general (van Baaren *et al*. 2004). We seem to be learning, not so much that this is a good person, but that the world is a good place. This unconscious mirroring can be seen as a consequence of activity in the brain's mirror system. Simply observing someone else move activates the same movements in the observer. Indeed, it is difficult to make a movement different from the one you are observing (Kilner *et al*. 2003). All these effects that we have mentioned so far are automatic and unconscious. Neither the sender nor the receiver need be aware that they are exchanging signals. Indeed, the rapport associated with the chameleon effect may be destroyed if we become aware that we are being imitated (Lakin & Chartrand 2003). Instead, we may feel we are being mocked.

## 4. Social signals that convey information

We can use social signals to help us attain our goals. If I am looking for a drink at a reception I can use the density of people in different parts of the room or the direction of their movements as signals indicating the probable location of the drinks table. Most of the time we use such social signals emitted by people (our conspecifics). But we can also use such signals from species other than our own. We train dogs to point at quarry such as hares and game birds and the Romans famously used geese to warn them of danger. And it is not just us. Many species use signals from other species to help them achieve their goals (Danchin *et al*. 2004). The important aspect of these signals is that they are emitted by agents, rather than objects. It is therefore important to be able to detect agents.

### (a) Detecting agents

We use very simple cues for detecting agents. A basic distinction is between self-propelled objects and non-self-propelled objects. An infant perceives causality when the motion of a non-self-propelled object is changed by another object. He or she perceives intention when a self-propelled object changes motion (Premack 1990). Infants treat self-propelled objects as agents with goals (Luo & Baillargeon 2005). Another important sign of agency is contingent behaviour. Infants will treat an inanimate object as an agent having communicative abilities and goal-directed behaviour, if the object interacts contingently with them or another person (Johnson 2003). Adults also, even though they know they are observing inanimate objects, such as triangles moving on a screen, are irresistibly driven to interpret the movements of these objects in terms of goals and intentions (Heider & Simmel 1944). This detection and interpretation of agents from movements seems to depend upon an automatic and highly stimulus-driven perceptual system (Scholl & Tremoulet 2000).

## 5. Beyond stimulus–response psychology: goals and actions

More recently, psychologists have started to think that the interaction between the person and the environment should be described the other way round. Rather than starting from a stimulus in the environment, the starting point is inside me and concerns my goals. What is currently my most pressing goal? How can this goal be best achieved given my prior knowledge and the current context? On the basis of the answers to these questions, I perform an act upon the world (engineers call this the input). This act will cause new signals to strike my senses (engineers call this the output) and I will learn whether or not the act has brought me nearer to my goal. The difference between what I expected and what actually occurred is the error signal that drives the system and enables me to approach my goals (Sutton & Barto 1998). Within this framework also we can define subsets of processes with specifically social functions. In particular, we can define social goals. Social goals are shared goals and therefore involve at least two people. Shared goals are most obviously involved in joint action, when at least two people are required to perform a task or when a task can be performed better by two people than by one person on his own. Successful joint action benefits from communication and also from trust. A shared goal is also implied when one person works for the benefit of others. However, social goals can also be competitive, as when one person tries to deceive another.

### (a) Alignment in joint action

Various kinds of alignment between the two participants are essential for the prosecution of shared actions. We need a shared vocabulary so that we can communicate and shared goals so that we can engage in joint activities. Clark (1996) has called this common ground. This sharing must occur at many levels of representation. For example, we should share each other's perception of the world. The starting point for sharing the world that we perceive is to align the focus of our attention. This process is called joint attention and is typically achieved by pointing at an object. This leads to the triadic relationship in which two people focus their attention on the same object. Background and foreground in their two perceptual worlds are now aligned. The wish to share attention in this way can be observed in infants as young as 12 months (Tomasello *et al*. 2005). Many aspects of alignment are achieved by deliberate communication. The infant points at the object he or she wants. Adults verbally agree on the joint goal towards which their action is aimed. However, there are many aspects of alignment that occur automatically.

### (b) Automatic alignment of goals

When we perform a task with someone we develop a shared representation of the whole task even though we are only performing part of it. In one paradigm (Sebanz *et al*. 2003), a pair of participants performed a ‘go–nogo’ task, sitting along side each other. Even though no interpersonal coordination was required, each actor integrated the co-actor's alternative action into their own action planning. This resulted in an action selection conflict when a stimulus required a different action from each actor (e.g. a ‘nogo’ response from one actor and a ‘go’ response from the other; Tsai *et al*. 2006). This effect seems to be automatic.

For this sort of alignment, simple imitation of action enabled by the mirror system is not sufficient. Having shared goals does not always mean that we should mirror each other's actions (Sebanz *et al*. 2006). For example, when two people are carrying a heavy object, one may walk backwards, while the other walks forwards. This complementary form of control often enables a joint action to be more efficient than the same action performed by a single person (Reed *et al*. 2006).

Automatic processes during joint action have been studied most extensively in relation to spoken dialogue (e.g. Pickering & Garrod 2004). For example, speakers give largely unconscious eye gaze signals to control turn-taking in discourse (Hedge *et al*. 1978). Likewise, they use interjections like ‘ah’ and ‘um’ to signal, respectively, forthcoming smaller or larger delays in speaking so as to avoid premature interruption (Clark & Fox Tree 2002). Two speakers also become more similar in their use of syntax. Branigan *et al*. (2000) asked pairs of speakers to take turns in describing pictures to each other. One speaker was a confederate of the experimenter and produced descriptions that systematically varied in syntactic structure. This primed a similar syntactic structure in the other speaker's subsequent description.

All these signals, which so strongly affect our verbal interactions, are largely unconscious and their role often comes as a surprise when revealed by clever experiments. In the next section, I shall consider the deliberate use and interpretation of signals.

## 6. The interpretation of signals

### (a) Learning by observation and learning by instruction

Most of the cognitive processes I have discussed so far function without awareness. People show emotional responses to fearful faces even though they are not aware of having seen the face (Morris *et al*. 1999). People also show emotional responses to untrustworthy faces even when they are attending to some other aspect of the face such as sex (Winston *et al*. 2002). We have also seen that the automatic imitation that comprises the chameleon effect only works when the participants are unaware that they are being imitated (Lakin & Chartrand 2003). In all these examples, the participants are unaware that they are sending or receiving signals. Thus, many social processes can occur without conscious awareness. There is much less evidence, however, as to whether certain social processes cannot occur in the absence of awareness.

The one exception is the study by Olsson & Phelps (2004) on the learning of fear through instruction. We have already seen that people can learn associate fear with an unseen stimulus during classical Pavlovian conditioning and also by the observation of someone else being conditioned. People can also learn by instruction (Phelps *et al*. 2001), that is, by being told that the stimulus (e.g. a blue square) will be followed by a painful shock. However, this learning by instruction does not generate a response when the stimulus is unseen. My interpretation of this result is that, when learning by instruction, we learn that the blue square is a signal that means that a shock will soon be coming. We cannot extract the meaning of this signal when it is processed below the level of consciousness. Response to the subliminal signal of fear can only be learned through a more primitive process of long-term association.

### (b) Deliberate signalling and knowledge transfer

The same important distinction applies when we consider the sender of the signals. This is the contrast between signals that result from involuntary responses to the object and signals sent with deliberate communicative intent. For example, a mother might deliberately simulate fear she did not feel in order to keep the infant away from a dangerous object. However, in most cases, deliberate signals are not deceptive. When directed at infants, deliberate signals are usually intended to teach (Csibra & Gergely 2006). Teaching is a particular kind of knowledge transfer, i.e. transfer by instruction. It is distinctly different from knowledge transfer by observation. A mother can display her knowledge simply by engaging in some skilled activity. The infant can learn by observing this activity. Indeed, this may be the only way in which infant apes acquire knowledge from their mothers (e.g. Maestripieri *et al*. 2002). In teaching knowledge transfer by instruction, the mother explicitly demonstrates her knowledge and ensures that the infant is in a receptive state for acquiring this knowledge.

### (c) Ostention

A typical teaching scenario is as follows. The mother first establishes eye contact with the infant, the mother then looks at and points to an object and the mother names the object. The first signal in the process, the mother looking at the infant, is not just a means of attracting the infant's attention, but it is also an *ostensive* gesture (Sperber & Wilson 1995). An ostensive gesture indicates that the signal that follows will be a deliberate communication about something of relevance to the receiver, ‘I am about to tell you something useful’.

Infants show special sensitivity to the ‘ostensive’ cues that signal the teacher's communicative intention to manifest new and relevant knowledge about a referent object. This kind of signalling is what occurs when learning by instruction is intended. For example, the rapid learning of the names for things that occurs during infancy seems to depend upon the infant recognizing the referential intentions of other people (Bloom 2002). In other words, an infant remembers a name when he or she recognizes that the adult is deliberately naming the object for his or her benefit. An example of the role of ostensive gestures in teaching comes from a study where infants learned a novel action on an object. Infants rapidly learned to turn on a light by touching a box with their head (Meltzoff 1988) when demonstration of this action was preceded by eye contact. But hardly any infants learned to imitate this action if it was not preceded by eye contact (Király *et al*. 2004).

Apart from eye contact, having one's name called is a very common ostensive signal. Infants are very sensitive to their name being called from the age of 4.5 months (Mandel *et al*. 1995). Another ostensive signal is the use of ‘motherese’ when talking to infants. Infants pay more attention to motherese than to normal adult speech (Fernald 1985) because they know that motherese is directed at them.

There is some evidence that this special kind of learning through instruction may be uniquely human (Maestripieri *et al*. 2002). While apes can learn by observation, there is little evidence for deliberate instruction of the use and recognition of ostensive signals that instruction will be forthcoming. Learning through observation can certainly lead to the spread of knowledge through a group creating a form of culture, but this mechanism will be far less efficient than the one based on deliberate instruction.

### (d) What do we learn about the world from instructions?

An ostensive signal indicates that the signals that follow are instructions that will reveal something relevant to us about the world. But how do we know whether these signals are valid? It seems that our default assumption is that these signals will be valid. We know, however, that signallers may sometimes be mistaken or deliberately deceptive. Csibra & Gergely (2006) suggest that, at 14 months, infants assume that signals following an ostensive gesture (i.e. instructions) are always valid. As a result, they combine the information from different sources to get a best estimate of what the referent object is really like. They do not recognize that different signallers may have different attitudes to the same object. By 18 months, infants recognize that different people have different attitudes to the same object. This is the same age at which infants begin to show an understanding of pretence (Friedman & Leslie 2007), recognizing that their mother has a special attitude to a banana, by pretending it is a telephone.

But once we realize that different signallers have different attitudes to objects, how do we decide which is the ‘correct’ attitude? However we make this decision, the mere fact that we have made it brings in all sorts of interesting social processes. Does the correct attitude depend upon the context? Are some people privileged signallers whose instructions are always treated as valid? In the early stages of infancy, the mother usually has this privileged status. Is there a standard or normal attitude to objects from which a few people deviate? This is why we can refer to some people as phobics because their attitude to objects (e.g. birds) is non-standard. Do we define out-groups as people with systematically different attitudes to objects from us?

The point I am making here is that, when we acquire knowledge from signals deliberately intended to instruct, we are entering the world of a much richer culture than can be obtained by learning through observation. It is this ability to deliberately share knowledge that makes the human mind unique. The cognitive essence of this ability is to recognize that certain signals are deliberately emitted and intended to instruct. This kind of cognition is sometimes called meta-cognition. It requires that we reflect on our own cognition, in this case the process of expressing and receiving signals. Meta-cognition is intimately associated with self-consciousness.

These signals upon which we can reflect are not restricted to vocalizations or gestures. Marks and arrangements of inanimate objects can also be used as deliberate signals. In this way, material becomes part of culture. Perhaps it is this ability to reflect upon our own signals that provided the basis for the extraordinary achievements of the human race during the last few thousand years. This development did not depend upon changes in the basic cognitive apparatus present in the human brain, but on the knowledge acquired by others and passed onto us by deliberate instruction.
